# Molecular Design of Luminescent Complexes of Eu(III): What Can We Learn from the Ligands

**DOI:** 10.3390/molecules28104113

**Published:** 2023-05-16

**Authors:** Julia Romanova, Rumen Lyapchev, Mihail Kolarski, Martin Tsvetkov, Denitsa Elenkova, Bernd Morgenstern, Joana Zaharieva

**Affiliations:** 1 Faculty of Chemistry and Pharmacy, Sofia University, James Bourchier 1 Blvd., 1164 Sofia, Bulgaria; ohrl@chem.uni-sofia.bg (R.L.); mkolarski@uni-sofia.bg (M.K.); nhde@chem.uni-sofia.bg (D.E.); 2 Department of Chemistry, Saarland University, 66123 Saarbrücken, Germany; bernd.morgenstern@uni-saarland.de

**Keywords:** lanthanoids, bidentate ligands, DFT/TD-DFT and TDA, antenna effect, energy transfer

## Abstract

The luminescent metal-organic complexes of rare earth metals are advanced materials with wide application potential in chemistry, biology, and medicine. The luminescence of these materials is due to a rare photophysical phenomenon called antenna effect, in which the excited ligand transmits its energy to the emitting levels of the metal. However, despite the attractive photophysical properties and the intriguing from a fundamental point of view antenna effect, the theoretical molecular design of new luminescent metal-organic complexes of rare earth metals is relatively limited. Our computational study aims to contribute in this direction, and we model the excited state properties of four new phenanthroline-based complexes of Eu(III) using the TD-DFT/TDA approach. The general formula of the complexes is EuL_2_A_3_, where L is a phenanthroline with –2–CH_3_O–C_6_H_4_, –2–HO–C_6_H_4_, –C_6_H_5_ or –O–C_6_H_5_ substituent at position 2 and A is Cl^−^ or NO_3_^−^. The antenna effect in all newly proposed complexes is estimated as viable and is expected to possess luminescent properties. The relationship between the electronic properties of the isolated ligands and the luminescent properties of the complexes is explored in detail. Qualitative and quantitative models are derived to interpret the ligand-to-complex relation, and the results are benchmarked with respect to available experimental data. Based on the derived model and common molecular design criteria for efficient antenna ligands, we choose phenanthroline with –O–C_6_H_5_ substituent to perform complexation with Eu(III) in the presence of NO_3_¯. Experimental results for the newly synthesized Eu(III) complex are reported with a luminescent quantum yield of about 24% in acetonitrile. The study demonstrates the potential of low-cost computational models for discovering metal-organic luminescent materials.

## 1. Introduction

The main power of the theoretical molecular design is that it can predict the properties of new advanced materials [1]. The theoretical molecular design saves time, allows strategic planning of the chemical experiments, minimizes the financial expenses, and gives a fundamental understanding of key structure–properties relationships, which determine the material performance. Despite all these indisputable advantages, application areas still exist, in which the use of the theoretical molecular design remains below its full potential. This is due to some limitations in the contemporary computational methods for materials modeling. Therefore, theoreticians constantly rush to develop new, more sophisticated approaches and tools, which can predict the material properties quickly and efficiently without the need for experimental input [2,3]. Once a new approach is approved, the theoretical molecular design becomes a main actor in the materials discovery. 

A good example of a field in which the predicting power of the theoretical molecular design remains limited is one of the metal-organic luminescent complexes of rare earth elements [4,5]. From an application point of view, these luminescent complexes are promising materials that can serve as chemical sensors [6,7], as photoactive compounds in organic light-emitting diodes [8], and even as antitumor drugs [9] and bioprobes [10,11]. From a fundamental point of view, the luminescent properties of metal-organic complexes of rare earth elements originate from an interesting and relatively rare photophysical phenomenon called the antenna effect [12]. The antenna effect is a consequence of the following cascade events within the complex: (1) the light photons are initially absorbed by the organic ligand, (2) as a result, the organic ligand is promoted to its lowest in energy singlet excited state and this excitation remains localized within the organic ligand, (3) the singlet excited state is afterwards transformed into a localized triplet excited state due to an efficient intersystem crossing process in the presence of a heavy atom, and (4) the triplet excited state of the organic ligand transfers its energy to the emitting levels of the rare earth ion, resulting in its luminescence. It is demonstrated that the antenna effect could happen only if the energy of the first excited triplet state of the ligand is at least 0.3 eV above the energy of the emitting levels of the rare earth metal ions [13]. A smaller energy difference, on the other hand, is also possible, but in this case, the rate of the reverse metal-to-ligand energy transfer is high, and hence the luminescent quantum yield is low. Therefore, the relative energy difference between the first excited triplet state of the ligand and the emitting level of the rare earth metal ion is a key quantity when aiming to predict the luminescent behavior of a new complex. 

One obstacle to the theoretical molecular design of new rare earth metal complexes is their ambiguous structure [14,15,16]. To satisfy the high coordination numbers typical for rare earth ions, up to 12, lanthanoids can form complexes with metal-to-antennae ligands ratios of 1:1, 1:2, 1:3, etc. It can also accommodate charged low molecular weight ligands and solvent molecules. Moreover, even for a fixed elemental composition of the complex, the conformational space is quite large. Such conformational freedom substantially enlarges the number of the structures, which must be simulated to predict the luminescent behavior of the compounds. Therefore, most of the existing theoretical investigations on rare earth complexes use crystallographic data as input, i.e., they rather explain the experimental luminescent behavior of the complexes than predict it. Another obstacle for the theoretical molecular design of new rare earth coordination compounds is that it requires optimization of triplet excited states. Because of the size of the complexes, such calculations are usually feasible with the broadly used time-dependent density functional theory (TD-DFT) [17]. However, it has been demonstrated that triplet states can be unstable in the framework of the TD-DFT [18], which will directly affect the energy difference used to predict the luminescent behavior of a new rare earth metal complex. Therefore, a preliminary benchmark on the energies of the excited triplet state concerning experimental data is usually required. And finally, another obstacle arises from the fact that in the real material the interactions of the rare earth metal ion with the environment (solvents, etc.) may suppress the photoluminescent response [19]. Such undesired effects can be alleviated by combining molecular dynamics and quantum chemical simulations, however, the large size of the systems limits the use of the hybrid approach in practice.

**Figure 1:**
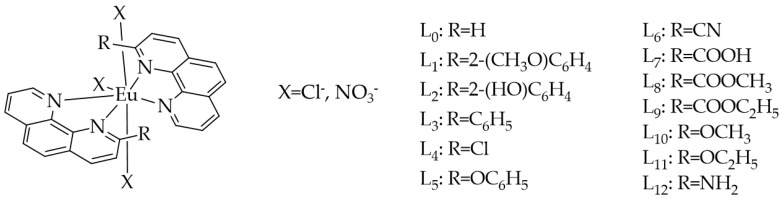
Structure of the ligands and the complexes simulated in this work: the complexes with L0, L4, and L6–L12 are available in the literature [20,22], and L1–L4 are newly designed.

Here, we demonstrate the predictive power of the theoretical molecular design for discovering new antenna ligands and luminescent rare earth metal complexes. We designed four new complexes with Eu^3+^, which are expected to have luminescent properties. The investigated Eu^3+^ complexes are structurally similar and synthetically feasible, and all contain phenanthroline-based antenna ligands (Figure 1). The general formula of the complexes is EuL_2_A_3_, where L is a substituted phenanthroline, such as L1 (–2–CH_3_O–C_6_H_4_), L2 (–2–HO–C_6_H_4_), L3(–C_6_H_5_) and L5 (–O–C_6_H_5_) and A is Cl^−^ or NO_3_^−^. The substituents are selected to vary in donor–acceptor strength, which will affect the antenna potential of the ligand but also, in experimental conditions, will impact the luminescence quantum yield, the solubility, and the crystal packing of the complexes. The structural similarity between the compounds allows us to approximate the input geometry and the ligand-to-metal ratios from existing crystallographic data on other phenanthroline-based complexes. To estimate the luminescent potential of the newly proposed compounds in a qualitative aspect, the set also comprises some already well-known antenna ligands like unsubstituted phenanthroline (L0) [20,21] and -2-Cl-substituted phenanthroline (L4) [22], as well as the corresponding experimentally characterized luminescent complexes as Eu(L0)_2_(NO_3_)_3_ and Eu(L4)_2_(NO_3_)_3_. Our computational study is performed with the TD-DFT method within the Tamm-Dancoff (TDA) approximation [23] and the ωB97xD functional [24]. It was already demonstrated that this theoretical approach successfully explores the structure and the photophysical properties of complexes containing rare earth metals because it reduces the problems arising from triplet instability [25,26]. The novelty of our computational study is that it reveals a simple quantitative relationship between the HOMO-LUMO gap of the isolated phenanthroline ligands and the excitation energy to the first triplet excited state of the complexes, which allows quick computational prediction of the luminescent properties of similar compounds. Finally, to confirm the computational model, we provide preliminary experimental results on the new metal-organic complex—Eu(L5)_2_(NO_3_)_3_, characterized by a luminescent quantum yield of about 24% in solution.

## 2. Results and Discussion

The TD-DFT/TDA results for the ligands and the newly designed complexes are represented in Table 1. Based on these calculations, we can estimate both the adiabatic and vertical emission energies (respectively wavelengths). For the goals of the present work, we should mainly discuss the adiabatic emission energies because the antenna transfer takes place after the excited state geometry relaxation occurs. According to available experimental data, the emitting level of Eu(III)—^5^D_0_ is located at 2.14 eV [27]. Therefore, the ligands, which can serve as antennae for the Eu(III), should possess a triplet state (adiabatic T_1_→S_0_ emission) located at 2.34 eV or above. 

The results for the isolated ligands obtained with the ωB97XD functional reveal adiabatic emission energies span the range of 2.77–2.93 eV. Therefore, in agreement with the experiment, this functional correctly predicts that the phenanthroline (L0) [20] and Cl-substituted phenanthroline (L4) [22] are suitable antennae for the Eu(III) ion. Moreover, it also suggests that all phenanthroline derivatives (L1–L3 and L5) investigated here are appropriate antennae when aiming to design europium-based luminescent complexes. The same qualitative conclusions can be made by looking into the ωB97XD results for the corresponding complexes. A quantitative difference, however, exists–the adiabatic emission energies for the complexes are systematically predicted at lower energies (0.04–0.13 eV) with respect to the adiabatic emission energies in the isolated ligands. Such a systematic quantitative difference can be interpreted as stabilization of the triplet excited state of the ligand in the Eu(III)/complex environment, and it is also consistent with the experimental measurements for phenanthroline and Eu(L0)_2_(NO_3_)_3_ [20,28]. A quantitative comparison with experimental data suggests that the ωB97XD functional overestimates the T_1_→S_0_ emission energy by 0.18 eV for the phenanthroline (L0) and by 0.22 eV for the Eu(L0)_2_(NO_3_)_3_ complex, which is a quite good agrеement bearing in mind that the calculated values are without a zero point vibrational energy correction. Even if we consider 0.18 eV/0.22 eV as a sign assigned errors for the theoretically estimated energy of the triplet excited state, all L0–L5 ligands are predicted to act as antennae for Eu(III). 

We also established good qualitative agreement between the electronic and optical properties of the isolated ligands and the energy of the triplet state in the corresponding complexes (Table 1 and Table 2). Such a conclusion is very helpful for molecular design purposes because it does not require knowledge of the stoichiometry and/or the conformation of the complex. Table 2 summarizes the ωB97XD results for the highest occupied molecular orbital HOMO, the lowest unoccupied molecular orbital LUMO, and the HOMO-LUMO gap of the isolated ligands. The substituents with heteroatoms (all except –C_6_H_5_) possess a negative inductive effect and a positive π-mesomeric effect with respect to the unsubstituted phenanthroline. Therefore, when attached to phenanthroline, these substituents are expected to increase the energy of the π-type HOMOs, as well as to decrease the HOMO-LUMO gap and the energy of the triplet excited state. This is exactly the observed trend for the –2–CH_3_O–C_6_H_4_, –2–HO–C_6_H_4,_ and –O–C_6_H_5_ functional groups. In this respect, the –Cl substituent is quite different because of the very unbalanced situation between its negative inductive effect and positive π-mesomeric effect. The positive π-mesomeric effect of the –Cl atom is indeed very weak or even suppressed by the high electronegativity of the atom, i.e., its prominent negative inductive effect amplified by the proximity of the pyridinic nitrogen atom. As a result, the –Cl substituent is expected to act as an acceptor rather than a donor. Therefore, adding a –Cl atom decreases both HOMO and LUMO. The average resultant effect of the functionalization with –Cl is the conservation of the HOMO-LUMO gap and of the energy of the triplet excited state with respect to the pure phenanthroline. The –C_6_H_5_ substituent is also a specific case in this series since it should possess both positive inductive and mesomeric effects concerning the unfunctionalized phenanthroline. As a result, the introduction of the phenyl group (1) decreases the energy of the LUMO, (2) increases the energy of the HOMO, (3) shrinks the HOMO-LUMO gap, and (4) decreases the energy of the excited triplet state by 0.16 eV with respect to the unsubstituted ligand.

**Figure 2:**
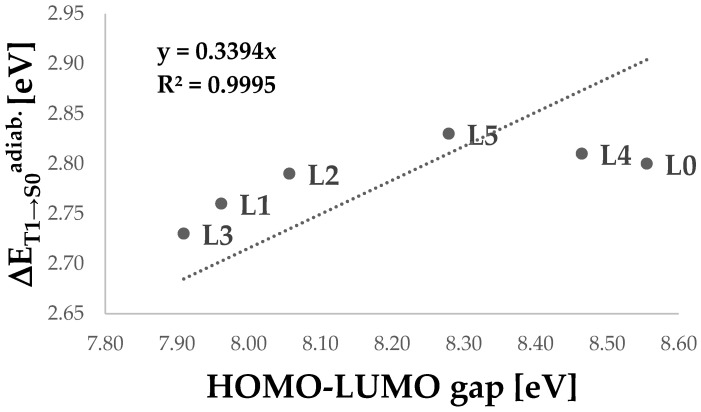
Relationship between the adiabatic energies of the first excited triplet state ∆E_T1→S0_^adiab.^ [eV] of the complexes and the HOMO-LUMO gap of the isolated ligands. The results are obtained with the ωB97xD functional. The correlation coefficient R^2^ and the equation of the linear fit are displayed, where x is the HOMO-LUMO gap in eV and y is the ∆E_T1→S0_^adiab.^ in eV.

Besides the qualitative agreement, we found a good quantitative relationship between the adiabatic energies of the first excited triplet state ∆E_T1→S0_^adiab.^ [eV] of the complexes and the HOMO-LUMO gap of the isolated ligands (Table 2). Both quantities can be related with a linear equation of type (Figure 2):

∆E_T1→S0_^adiab.^ (complex) [eV] = 0.3394 HOMO-LUMO gap (ligand) [eV] (1)

with quite a high coefficient of determination, R^2^ = 0.9995. From a molecular design perspective, this is a very useful model because the computation of the HOMO-LUMO gap of a ligand is a quick calculation compared to the excited state optimization of complex compounds. To test the applicability of Equation (1), we took seven phenanthroline-based ligands, which are ‘unknown’ for the model (L6–L12, Figure 1) and for whose complexes with formula EuL_2_(NO_3_)_3_ they are available experimental data in the literature (Table 3) [22]. The seven ligands differ in the substituent at position 2 in the phenanthroline system: L6 (-CN), L7 (-COOH), L8 (-COOCH_3_), L9 (-COOC_2_H_5_), L10 (-OCH_3_), L11 (-OC_2_H_5_) and L12 (-NH_2_). According to Equation (1), all those ligands possess high enough energy in triplet excited state (Table 3). Based on the principle for energy level alignment, the antenna transfer to Eu(III) is expected to occur. In this respect, there is a very good agreement between the experimental observations and our theoretical predictions. At first sight, there is only one exception for the complex bearing L12. However, a careful examination of the reported experimental data reveals that the authors measured a suitable triplet excited state at 2.54 eV for L12 and explained the absence of luminescence in the Eu(L12)_2_(NO_3_)_3_ complex with the presence of a quenching mechanism [22]. The latter is indeed not taken into account in our model. Moreover, if we correct the predicted values for the ∆E_T1→S0_^adiab.^ in the complexes by 0.22 eV (the error in the calculation), we obtain 2.58 eV for the triplet excited state of the complex bearing L12, i.e., our low-cost theoretical model is just 0.04 eV above the experimentally measured value of 2.54 eV. This again confirms the molecular design potential of the derived model relationship.

Since in most cases the excited state properties of the isolated ligand determine the photophysical behavior of the lanthanide complexes, our computational approach can be used to derive simple models for discovering luminescent complexes of rare earth ions with non-phenanthroline based antennae. Moreover, such a model can be improved further to consider other factors which have important effects on the luminescent quantum yeild, such as, for example, the presence of substituents with undesired quenching effects, etc. However, the elaboration of the computational model in this direction should be done with respect to considerably large experimental datasets obtained in equal experimental conditions and with identical apparatus. Although this is out of the scope of this work, we believe that our results are encouraging and will serve as an inspiration in the field.

Finally, to obtain an empirical confirmation of the derived computational model, we performed synthesis and characterization of the new metal-organic complex with L5—Eu(L5)_2_(NO_3_)_3_. The choice of the L5 ligand for the preliminary experimental study was based on the model but also on some common molecular design criteria for efficient antennae. Namely, we chose L5 because its structure does not contain -CH_3_ and -OH groups, known as luminescence quenchers [29,30]. 

**Figure 3:**
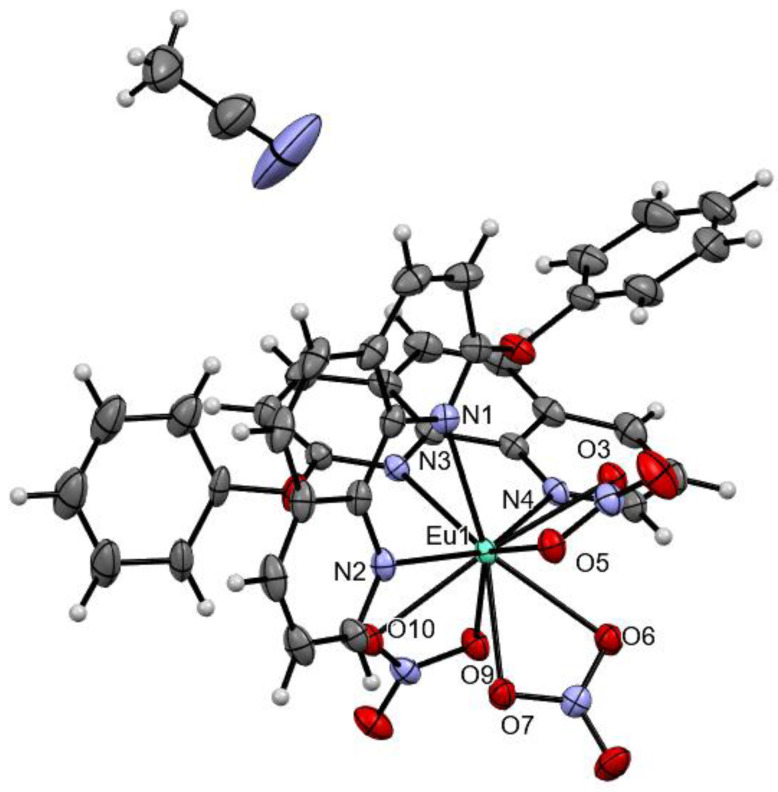
Molecular structure of Eu(L5)_2_(NO_3_)_3_ with displacement ellipsoids at the 50% probability.

The molecular structure of the Eu(L5)_2_(NO_3_)_3_ complex is represented in Figure 3. The complex crystalizes in the triclinic *P*$\bar{1}$ space group where the central Eu(III) is ten coordinated by four N-atoms from the 2-phenoxy-1,10-phenanthroline ligands. The coordination sphere is completed by three nitrate anions linked in bidentate fashion (Table 4). The bond lengths between Eu(III) and N-atoms are in the range of 2.5645(15) Å–2.5919(16) Å, while the distance between the central metal ion and the oxygen atoms from the nitrate anions is in the range of 2.4955(14)–2.5455(14) Å (Supplementary Materials, Table S1). The shortest Eu(III)---Eu(III) distance is 10.3835(5) Å, which is a prerequisite for an efficient luminescent material since the concentration quenching will be minimized [31]. 

**Figure 4:**
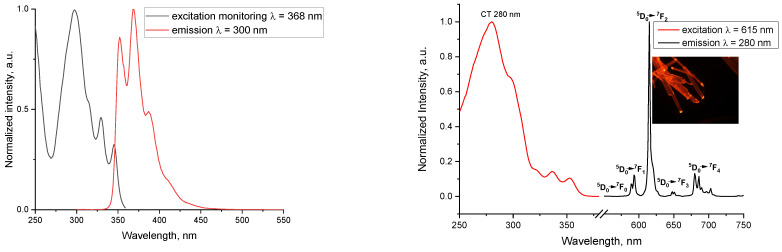
Excitation and emission spectra in acetonitrile (1 × 10^−6^ M solutions): the 2-phenoxy-1,10-phenanthroline (L5) ligand (**left**) and Eu(L5)_2_(NO_3_)_3_ complex (**right**).

The excitation and emission spectra of the L5 ligand in acetonitrile (1 × 10^−6^ M) are shown in Figure 4. As expected, the fluorescence of the isolated ligand is short-lived and intense, which is typical behavior for the 1, 10-phenanthroline system in polar solvents. The broad emission with a well-defined maximum at 368 nm corresponds to the lowest singlet state with π−π* origin [33].

The excitation spectrum of the Eu(L5)_2_(NO_3_)_3_ complex (Figure 4) has similar features to the excitation spectrum of the isolated L5 ligand. The major difference is in the position of the absorption/excitation maximum, which is slightly blue-shifted with approximately 20 nm and appears at 280 nm in the complex. This is a clear indication of the successful complexation and the stability of the complex even at 1 × 10^−6^ M concentration. The emission spectrum of the Eu(L5)_2_(NO_3_)_3_ complex shows the characteristic europium-centered fluorescence due to ^5^D_0_ → ^7^F_J_ (J = 0–4) transitions. The hypersensitive electric dipole ^5^D_0_ → ^7^F_2_ transition is the most intense one and represents 64.2% of the whole emission of the complex. This, in addition to the fact that the ^5^D_0_ → ^7^F_0_ transition is only 0.2% of the whole emission spectrum, is a clear indication of the high symmetry of the complex [28]. The measured emission quantum yield in acetonitrile is 24.15% ± 0.36%, which is significantly higher than the results obtained previously for other Eu(III) complexes, synthesized in our group with 2-(phenylethynyl)-1,10-phenanthroline [25]. Such improvement of the quantum yield can be explained by the higher in energy triplet excited state in the case of the L5 ligand with respect to the previously reported antenna—2-(phenylethynyl)-1,10-phenanthroline. Comparison with the results of Pan et al. for other phenanthroline-based Eu(III) complexes in acetonitrile suggests that the quantum yield for the Eu(L5)_2_(NO_3_)_3_ complex is similar or higher. However, it is important to note that we measured absolute fluorescence quantum yield with only ±0.36% error, while Pan et al. reported relative fluorescence quantum yield with an error of 15% [34]. Besides these particularities in the experimental measurements, all these evaluations confirm the suitability of the L5 ligand to act as an effective antenna for Eu(III) and the applicability of the model.

**Figure 5:**
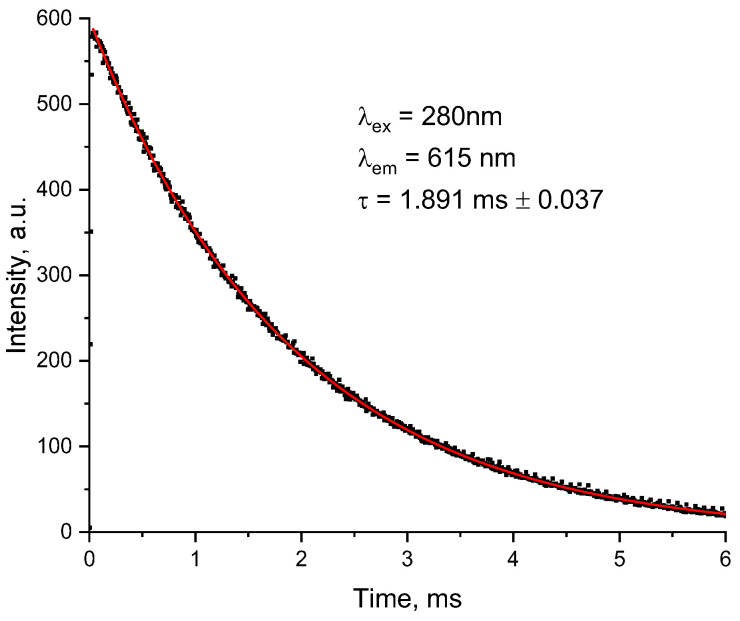
Luminescence lifetime decay profile and single exponential fit of Eu(L5)_2_(NO_3_)_3_.

The fluorescence lifetime of Eu(L5)_2_(NO_3_)_3_ was measured by monitoring the ^5^D_0_ → ^7^F_2_ transition (615 nm), and the decay curves were fitted by using a single exponential function (R^2^ = 0.99957), which suggests a single major luminescence species in the complex (Figure 5) [35]. The experimentally measured fluorescence lifetime (τ = 1.891 ms) is almost 2 times higher than the previously reported Eu(III) complexes with substituted 1,10-phenanthroline complexes in solid state and acetonitrile [22,35].

**Figure 6:**
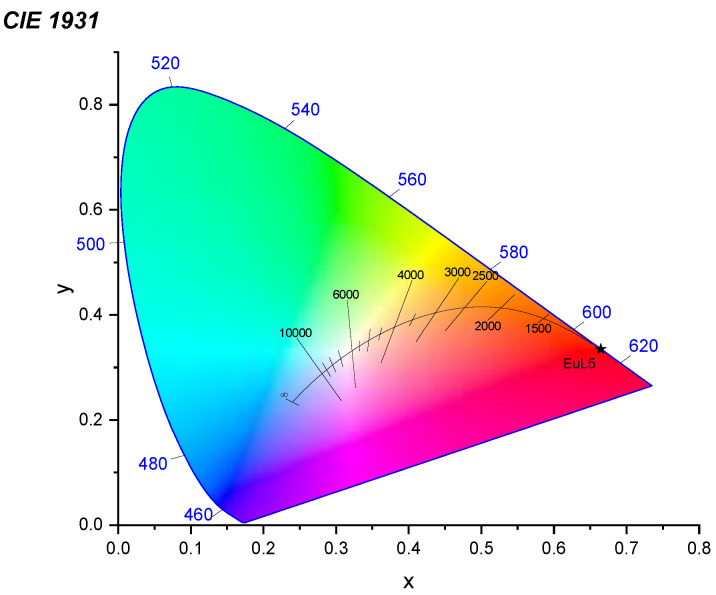
CIE 1931 X-Y color coordinates for Eu(L5)_2_(NO_3_)_3_.

The obtained CIE 1931 X-Y color coordinates (Figure 6) show that the sample emission lies in the orange-red region with coordinates (0.6645, 0.3350). Based on these coordinates, the calculated color purity is approximately 99% (for the dominant 609.7 nm wavelength).

## 3. Materials and Methods

### 3.1. Computational Protocol

The ground states of the isolated ligands and their corresponding complexes of type Eu(L)_2_A_3_ (where A = Cl^−^ or NO_3_^−^) are optimized with the ωB97xD functional [24]. The initial atomic coordinates used for geometry optimizations of the complexes are taken from the crystallographic data for structurally similar Eu(III)-complexes synthesized recently by our group [25]. The Pople’s 6-31G* basis set is used for all non-metal elements. For europium, the MWB52 basis sets and effective core potentials are applied to consider the relativistic effects [36]. After the ground state geometry optimization, a frequency analysis is performed to prove that the obtained structures represent minima on the potential energy surface.

The adiabatic energies of the singlet and triplet excited states were calculated using the TD-DFT method within the Tamm-Dancoff (TDA) approximation [23]. The TDA approximation works well for rare-earths antennae complexes since it prevents triplet instability problems [18]. The excited state geometry optimization was performed with the ωB97XD functionals in combination with the same basis sets and pseudopotentials as in the ground state geometry optimization. All calculations were done in a vacuum with the Gaussian 09 program [37].

### 3.2. Preparation of the L5 Ligand 

**Scheme 1:**
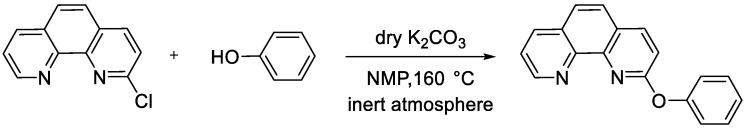
Synthesis of L5.

The synthesis of L5 is presented on Scheme 1 and represents the nucleophilic substitution of the chlorine atom in position C-2 of the phenanthroline system.

The 2-chloro-1,10-phenanthroline [38] is reacted with a slight excess of phenol [39] in N-methylpyrrolidone (NMP) media in the presence of potassium carbonate. Ligand L5 was isolated with a yield of 80%. Here, it is important to note that initially, as suggested by other authors, the same synthetic procedure with dimethylformamide as solvent [40] was applied; however, a side byproduct was observed. Thus, making it difficult to be removed from the target compound 2-phenoxy-1,10-phenanthroline.

The N-methylpyrrolidone was purchased from Merck, and phenol and K_2_CO_3_ were purchased from a local supplier. N-methylpyrrolidone and phenol were used as received. K_2_CO_3_ was dried by heating in an open vessel and then finely powdered in a mortar.

**Figure 7:**
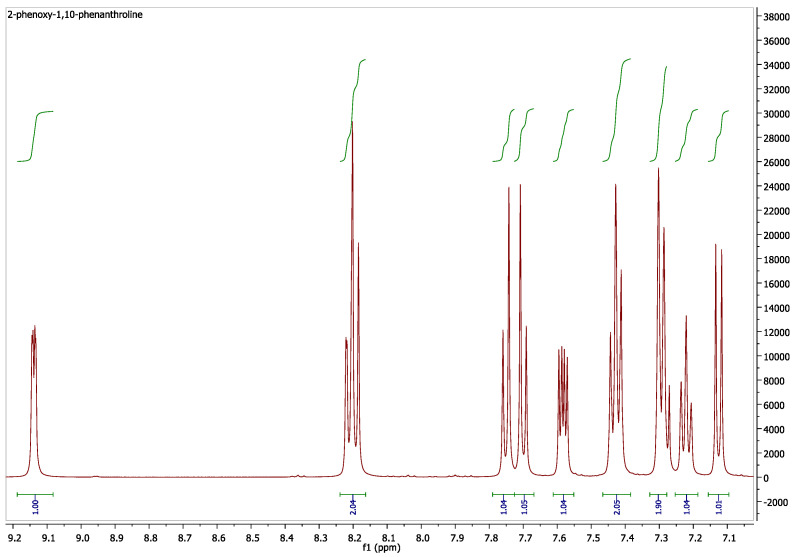
^1^H NMR of L5.

In a Schlenk flask were mixed 2-chloro-1,10-phenanthroline (1.3 g, 6.06 mmol, 1 eqv), phenol (0.888 g, 9.44 mmol, 1.56 eqv), finely powdered anhydrous K_2_CO_3_ (2.511 g, 18.17 mmol, 3 eqv) and 10 mL NMP. Air in the vessel was evacuated under vacuum and replaced with argon. The reaction was monitored with thin-layer chromatography. The flask was heated at 160 °C untill full consumption of the starting 2-chlorophenanthroline (22 h). After that, NMP was removed under reduced pressure, and the residue was dissolved in 150 mL dichloromethane (DCM). The resulting solution was washed with distilled water (3 × 30mL), aqueous NaOH (1 mol/L, 1 × 25 mL), and again with distilled water (1 × 25 mL). After drying (Na_2_SO_4_), the solvent was removed under reduced pressure. The crude product was purified using flash column chromatography on silica, eluting with hexanes/DCM, DCM, and DCM/methanol, and then on neutral alumina, eluting with hexanes/DCM. The yield is 1.317 g (80%), and at this stage, the sample represents a pale yellow powder. After recrystallization from acetonitrile white crystals were obtained. ^1^H NMR of L5 is represented on Figure 7.

**^1^H NMR** δ 9.14 (dd, J = 4.3, 1.7 Hz, 1H, ***H9****-phenanthroline*), 8.22 (dd, J = 8.16, 1.74 Hz, 1H, ***H7****-phenanthroline*), 8.20 (d, J = 8.6 Hz, 1H, ***H4****-phenanthroline*), 7.76 (d, J = 8.7 Hz, 1H, ***H5****-phenanthroline*), 7.71 (d, J = 8.7 Hz, 1H, ***H6****-phenanthroline*), 7.59 (dd, J = 8.1, 4.3 Hz, 1H, ***H8****-phenanthroline*), 7.47–7.41 (m, 2H, H3, ***H5****-phenyl*), 7.30 (dd, J = 8.5, 0.9 Hz, 2H, H2, ***H6****-phenyl*), 7.23 (tt, J = 7.4, 1.03 Hz, 1H, ***H4****-phenyl*), 7.14 (d, J = 8.6 Hz, 1H, ***H3****-phenanthroline*).

**^13^C NMR** δ 162.50 (s, ***C2****-phenanthroline*), 154.73 (s, ***^4^C1****-phenyl*), 150.17 (s, ***C9****-phenanthroline*), 145.34 (s, ***^4^C10a****-phenanthroline*), 145.08 (s, ***^4^C10b****-phenanthroline*), 139.89 (s, ***C4****-phenanthroline*), 135.90 (s, ***C7****-phenanthroline*), 129.92 (s, ***C3,C5****-phenyl*), 129.10 (s, ***^4^C6a****-phenanthroline*), 125.87 (s, ***C5****-phenanthroline*), 125.51 (s, ***^4^C4a****-phenanthroline*), 124.81 (s, ***C6****-phenanthroline*), 124.66 (s, ***C4****-phenyl*), 122.95 (s, ***C8****-phenanthroline*), 120.74 (s, ***C2,C6****-phenyl*), 112.68 (s, ***C3****-phenanthroline*). R_f_ TLC 0.53 (neutral Al_2_O_3_, DCM), 0.20 (silica 20:1 DCM/methanol). m.p. 160.2–161.7 °C.

### 3.3. Synthesis of the Eu(L5)_2_(NO_3_)_3_ Complexes

The complex was prepared by the procedure described in [25]. Eu(NO_3_)_3_∙5H_2_O and the ligand were dissolved in acetonitrile and mixed at 80 °C. Afterward, the mixture was heated to 80 °C and stirred for 7 h. The molar ratio of metal to ligand is 1:2. 

The precipitate was filtrated, washed with acetonitrile, and dried at room temperature. Out of the powder sample obtained, single crystals of the complex in acetonitrile media were formed by slow evaporation. 

### 3.4. Characterization of L5 and Eu(L5)_2_(NO_3_)_3_

X-ray diffraction measurements were made at 133 K on a Bruker D8 Venture diffractometer with a microfocus sealed tube and a Photon II detector using graphite monochromated Mo-Kα radiation (λ = 0.71073 Å). NMR spectra were recorded on a Bruker Avance III 500 spectrometer at 500 MHz for ^1^H and 125.7 MHz for ^13^C. Lifetime measurements and photoluminescence measurements of the complexes were made on a Cary Eclipse spectrometer with a xenon lamp as an excitation source, as well as on an N-400M fluorescent microscope. The quantum yields and photoluminescence measurements are performed at FluoroLog3-22, Horiba JobinYvon equipped with an integration sphere. 

The crystal structure was solved by direct methods using SHELXT [41] and was refined by full-matrix least-squares calculations on F2 (SHELXL2018 [42]) in the graphical user interface Shelxle [43]. The molecular structure is represented using Mercury v4.0 software [44]. The CIE 1931 chromatograms were obtained using the LED ColorCalculator v7.77 [45].

## 4. Conclusions

A total of four new Eu(III) complexes with potential luminescent properties are theoretically designed using the TD-DFT/TDA approach and the ωB97xD functional. The general formula of the complexes is EuL_2_A_3_, where L is a phenanthroline-based ligand, which serves as an antenna for energy transfer to the emitting levels of the metal, and A is Cl^−^ or NO_3_^−^. The newly designed complexes differ in the substituent at position 2 in the phenanthroline system: L1 (-2-CH_3_O-C_6_H_4_), L2 (-2-HO-C_6_H_4_), L3(-C_6_H_5_), and L5 (-O-C_6_H_5_). The computational strategy is benchmarked with respect to structurally similar existing phenanthroline-based complexes with L0 (-H) and L4 (-Cl). In addition to the complexes, the properties of the ligands are also simulated. The luminescent properties of the complexes are predicted based on the energy alignment between the first excited triplet state of the complexes/ligands and the emitting levels of Eu(III). The error in the calculated adiabatic energies of the first excited triplet state is 0.18 eV and 0.22 eV for the ligands and the complexes, respectively. We found good qualitative and quantitative agreement between the electronic properties of the isolated ligands and the energy of the key first excited triplet state of the complexes. Substituents with a positive π-mesomeric effect decrease the HOMO-LUMO gap and the energy of the triplet excited state, and this decrease depends on the inductive effects. The HOMO-LUMO gap and the energy of the triplet excited state decrease going from a positive to a strong negative inductive effect of the substituent in the phenanthroline system. In addition, a linear relationship between the energy of the HOMO-LUMO gap of the ligands and the energy of the triplet excited state is derived with a high determination coefficient (R^2^ = 0.9995). The model relationship is tested on experimentally characterized 2-substituted phenanthroline complexes of type EuL_2_NO_3_, where L6 (-CN), L7 (-COOH), L8 (-COOMe), L9 (-COOEt), L10 (-OMe), L11 (-OEt) and L12 (-NH_2_). In agreement with the experimental observation, the energy of the triplet excited state in all L6–L12 complexes is computationally estimated as suitable for energy transfer to the emitting levels of Eu(III). From a molecular design perspective, the possibility of building a model which relates the electronic properties of isolated ligands with the excited state properties of complexes is a promising result. To confirm the validity of the derived model, we report preliminary experimental results on the newly designed Eu(L5)_2_(NO_3_)_3_ complex with a luminescent quantum yield of about 24% in acetonitrile. The L5 ligand with –O–C_6_H_5_ substituent was selected based on the model and common molecular design criteria for efficient antenna ligands, such as the lack of functional groups acting as luminescence quenchers. We believe that similar computational models can also be developed for non-phenanthroline-based complexes of rare earth ions and that they can be improved further to consider other structural factors directly impacting the quantum yield. 

## Figures and Tables

**Table 1:** TD-DFT/TDA results for the first excited triplet state of the ligands (L0–L5) and the corresponding complexes calculated with different DFT functionals: adiabatic (∆E _T1→S0_^adiab.^ [eV]) and vertical emission energies (∆E _T1→S0_^vert.^ [eV]), adiabatic (λ _T1→S0_^adiab.^ [nm]) and vertical emission wavelengths (λ _T1→S0_^vert.^ [nm]). Where appropriate, the experimentally measured values are also reported.

∆E_T1→S0_^adiab.^ [eV] (λ _T1_→_S0_^adiab.^ [nm]) and ∆E_T1→S0_^vert.^ [eV] (λ _T1_→_S0_^vert^ [nm])			
B97XD			
Complex		Ligand	
Eu(L0)_2_Cl_3_	2.80^adiab.^(443) and 2.48 ^vert.^(499)	L0	2.93 ^adiab.^(423) and 2.46^vert.^(505) 2.75–2.74 ^exp.^ [28]
Eu(L0)_2_(NO_3_)_3_	2.80^adiab.^(444) and 2.48 ^vert.^(500) 2.58^exp.^ [20]		
Eu(L1)_2_Cl_3_	2.76^adiab.^(449) and 2.44 ^vert.^(508)	L1	2.85^adiab.^(435) and 2.40 ^vert.^(517)
Eu(L2)_2_Cl_3_	2.79^adiab.^(444) and 2.48 ^vert.^(500)	L2	2.86^adiab.^(434) and 2.40 ^vert.^(516)
Eu(L3)_2_Cl_3_	2.73^adiab.^(454) and 2.43 ^vert.^(511)	L3	2.77^adiab.^(447) and 2.36 ^vert.^(526)
Eu(L4)_2_(NO_3_)_3_	2.81^adiab.^(442) and 2.50 ^vert.^(497)	L4	2.93^adiab.^(423) and 2.46 ^vert.^(504)
Eu(L5)_2_(NO_3_)_3_	2.83^adiab.^(438) and 2.52 ^vert.^(493)	L5	2.92^adiab.^(425) and 2.46 ^vert.^(503)

**Table 2:** Energies of the HOMO and the LUMO and the HOMO-LUMO gap of the isolated ligands (L0–L5) calculated with the ωB97XD functional.

	E_HOMO_ [eV]	E_LUMO_ [eV]	HOMO-LUMO Gap [eV]
L0	−8.17	0.39	8.56
L1	−7.56	0.41	7.96
L2	−7.65	0.41	8.06
L3	−7.74	0.17	7.91
L4	−8.31	0.15	8.46
L5	−7.82	0.46	8.28

**Table 3:** Predicted luminescent behavior of Eu(III) complexes with seven ‘unknown’ for the model phenanthroline-based ligands (L6–12) [22] through Equation (1): HOMO-LUMO gaps for the isolated ligands obtained with the ωB97XD functional, predicted adiabatic energies for the first excited triplet state ∆E_T1→S0_^adiab.^ in Eu(III)-complexes, predicted adiabatic energies for the first excited triplet state ∆E_T1→S0_^adiab.^ in Eu(III)-complexes corrected with −0.22 eV, experimental luminescent response of complexes of type Eu(L)_2_(NO_3_)_3_.

Ligand	HOMO-LUMO Gap [eV]	Predicted ∆E_T1→S0_^adiab.^ [eV]	Predicted and Corrected ∆E_T1→S0_^adiab.^ [eV]	Luminescent Behavior
L6	8.17	2.77	2.55	yes
L7	8.20	2.78	2.56	yes
L8	8.23	2.79	2.57	yes
L9	8.24	2.80	2.58	yes
L10	8.24	2.80	2.58	yes
L11	8.24	2.80	2.58	yes
L12	8.24	2.80	2.58	no *

* The experimentally estimated ∆E_T1→S0_ in Ln(L)_2_(NO_3_)_3_ is 2.54 eV (20500 cm^−1^), i.e., the antenna effect is, in principle, possible but not observed due to a quenching mechanism [22].

**Table 4:** Crystallographic data for the Eu(L5)_2_(NO_3_)_3_ complex.

	Eu(L5)_2_(NO_3_)_3_
Crystal data	
Empirical formula	C_38_H_27_N_8_O_11_Eu
Molecular weight (g/mol)	923.63
Crystal system, space group	Triclinic, *P*$\bar{1}$
Temperature (K)	133(2)
*a*, *b*, *c* (Å)	10.3835(4), 11.4085(5), 16.0632(6)
*α*, *β*, *γ* (°)	92.860(2), 103.351(2), 95.062(2)
V, (Å^3^)	1839.35(13)
*Z*	2
Radiation type	Mo *Kα*
μ, (mm^−1^)	1.780
Crystal size (mm)	0.220 × 0.120 × 0.040
Data collection	
Diffractometer	Bruker D8 Venture
Absorption correction	Multi-scan (SADABS) [32]
T_min_, T_max_	0.6838, 0.7456
No. of measured, independent and observed [I > 2σ(I)] reflections	50299, 8799, 8306
R_int_	0.0456
(sin θ/λ)_max_, (Å^−1^)	0.639
Refinement	
R[F^2^ > 2σ(F^2^)], wR(F^2^), S	0.0196, 0.0487, 1.066
No. of reflections	8799
No. of restrains	282
No. of parameters	579
H-atom treatment	H-atom parameters constrained
Δρ_max_, Δρ_min_ (e Å^−3^)	0.393, −0.458

## Data Availability

Crystallographic data have been deposited and are freely available at Cambridge Crystallographic Data Center under the reference number: 2260915.

## Supplementary Materials

The following supporting information can be downloaded at: https://www.mdpi.com/article/10.3390/molecules28104113/s1, Table S1: Crystallographic data for the Eu(L5)_2_(NO_3_)_3_: selected bond lengths and bond angles.

## Sample Availability

Samples of the compounds in this article are not available from the authors.
