# Supplementary material for: Molecular Design of Luminescent Complexes of Eu(III): What Can We Learn from the Ligands
Source: Molecules. 2023 May 16;28(10):4113. doi: 10.3390/molecules28104113 (PMC10221419; doi:10.3390/molecules28104113)
Supplement: Supplementary file 1 [file molecules-28-04113-s001.zip › molecules-2361721-supplementary.pdf]

Table S1. Crystallographic data for the Eu(L5)2(NO3)3: selected bond lengths and bond angles.

**Selected bond lengths, Å**		**Selected bond angles, º**	
Eu(1)-O(7)	2.4954(14)	N(2)-Eu(1)-N(3)	97.28(5)
Eu(1)-O(5)	2.5007(14)	N(4)-Eu(1)-N(3)	63.36(5)
Eu(1)-O(6)	2.5095(13)	N(2)-Eu(1)-N(4)	158.42(5)
Eu(1)-O(3)	2.5153(13)	N(2)-Eu(1)-N(3)	97.28(5)
Eu(1)-O(10)	2.5439(13)	N(2)-Eu(1)-N(1)	63.98(5)
Eu(1)-O(9)	2.5455(13)	N(4)-Eu(1)-N(1)	98.73(5)
Eu(1)-N(2)	2.5644(14)	N(3)-Eu(1)-N(1)	70.60(5)
Eu(1)-N(4)	2.5823(15)		
Eu(1)-N(3)	2.5919(15)		
Eu(1)-N(1)	2.5919(15)		
